# Active Integrated Filters for RF-Photonic Channelizers

**DOI:** 10.3390/s110201297

**Published:** 2011-01-25

**Authors:** Amr El Nagdi, Ke Liu, Tim P. LaFave, Louis R. Hunt, Viswanath Ramakrishna, Mieczyslaw Dabkowski, Duncan L. MacFarlane, Marc P. Christensen

**Affiliations:** 1 Department of Electrical Engineering, University of Texas at Dallas, P.O. Box 830688, Richardson, TX 75083, USA; 2 Department of Mathematical Sciences, University of Texas at Dallas, P.O. Box 830688, Richardson, TX 75083, USA; 3 Department of Electrical Engineering, Southern Methodist University, P.O. Box 750338, Dallas, TX 75275-0338, USA

**Keywords:** photonic channelizer, active filters, four-port coupler, state space representation

## Abstract

A theoretical study of RF-photonic channelizers using four architectures formed by active integrated filters with tunable gains is presented. The integrated filters are enabled by two- and four-port nano-photonic couplers (NPCs). Lossless and three individual manufacturing cases with high transmission, high reflection, and symmetric couplers are assumed in the work. NPCs behavior is dependent upon the phenomenon of frustrated total internal reflection. Experimentally, photonic channelizers are fabricated in one single semiconductor chip on multi-quantum well epitaxial InP wafers using conventional microelectronics processing techniques. A state space modeling approach is used to derive the transfer functions and analyze the stability of these filters. The ability of adapting using the gains is demonstrated. Our simulation results indicate that the characteristic bandpass and notch filter responses of each structure are the basis of channelizer architectures, and optical gain may be used to adjust filter parameters to obtain a desired frequency magnitude response, especially in the range of 1–5 GHz for the chip with a coupler separation of ∼9 mm. Preliminarily, the measurement of spectral response shows enhancement of quality factor by using higher optical gains. The present compact active filters on an InP-based integrated photonic circuit hold the potential for a variety of channelizer applications. Compared to a pure RF channelizer, photonic channelizers may perform both channelization and down-conversion in an optical domain.

## 1. Introduction

RF photonics technology extending from coaxial cable replacement in RF communication links to signal processing in an optical domain, has recently led to higher efficiency, less complexity, and lower cost than conventional electronic systems, especially at high microwave and millimeter wave frequencies [1,2]. Channelization is a useful technique for simultaneously resolving multiple narrow frequency bands from a wideband RF spectrum used for communication and radar systems. Photonic channelization offers many advantages in processing ultra-wideband RF signals compared to pure electronic solutions, for example, large instantaneous bandwidth offered by photonics technology and cost saving of post-processing electronics as the channelization of broadband signals translating into intermediate frequencies [3]. On the other hand, frequency down-conversion may be realized by using optical heterodyne detection [4]. The technique mixes a channelized optical signal and an optical local oscillator signal by a photonic coupler. The outputs connect to photodetectors constituting the optical-to-electronic converters of sub-receivers. Strictly speaking, the integral down-conversion technique using photonic channelizers occurs partly in the optical and partly in the electronic domain.

Many optical channelizer approaches have been attempted. The optical filter is a key element for the realization of a photonic channelizer. Tunable frequency response filters are becoming strongly desired for exploiting the full bandwidth available. State of the art photonic channelizers are based on optical filter banks that are implemented via various filtering techniques, including free-space diffraction grating [3], Bragg-grating Fabry-Perot cavity [5–7], discrete element [8], and ring resonators [9]. However, these passive optical devices are not sufficiently flexible to be tuned or are limited by bulky optical implementation.

In this work, four different two-dimensional (2D) active filter architectures are proposed, which may all be considered building blocks for photonic channelizers. Gains are incorporated in these structures to reduce net loss and to provide tunability by emphasizing or de-emphasizing certain frequency components. In addition to gain elements, all four architectures consist of nano-photonic couplers (NPCs) that are interconnected by multi-quantum well (MQW) InP ridge waveguides. The architectures make use of two- and four-port couplers and differ by their respective the structural layout.

In the signal processing domain, three different functions are fundamental to filter operation. The first function is the ability to split signals into different paths or branches. Optically, this function is achieved by the use of photonic couplers. A coupler is capable of splitting different incoming signals into a number of different waveguides. Practically, splitting of the signal involves a different scaling factor for each output signal. For example, in the case of a two-port coupler, an incoming signal is split into two different waveguides with two different transmission and reflection scaling factors. The second function of a filter is its ability to combine or sum different combinations of incoming signals. Again, photonic couplers may accomplish this task by the coupling of different signals from different waveguides into a single waveguide. The resultant signal could be either a direct summation of the different signals combinations or cancellation among some combinations depending on the phase of incoming signals. The third function that filters provide is the introduction of time delay between summing and splitting nodes. A true time delay may be provided by length of waveguide between adjacent couplers. This may be realized by the inducing of semiconductor optical amplifiers (SOAs). SOAs not only provide device tunability but necessarily introduce true time delays [10,11]. Variations of the previous basic function characteristics change the filter’s spectral characteristics.

The couplers design method proposed here is based on a concept of frustrated total internal reflection, which achieves a compact and efficient way of controlling signal reflection and transmission coefficients [12,13]. Also, these couplers may be fabricated using conventional microelectronics processing techniques that make it more advantageous [14]. The types of couplers considered in the proposed architectures are 1 × 2 and 2 × 2 couplers. The 1 × 2 coupler simply splits an input signal into two components. Depending on coupler orientation in the waveguide, a coupler may produce either a right directed signal, *α*, or a left directed signal, *β*, in addition to a straight transmitted signal, *τ*. The 2 × 2 four-port coupler may support up to four input signals and produce four output signals for each input. At each coupler port, there is a reflected component, *ρ*, a transmitted component, *τ*, a right-directed component, *α*, and a left-directed component, *β* [15]. The varied manner in which these couplers may be arranged, yields very rich optical characteristics.

Section 2 illustrates network diagrams of the four photonic channelizer architectures. Section 3 presents how these channelizers are fabricated on InP-based wafers using conventional microelectronics processing techniques. Section 4 describes how the channelizers are modeled with a state space modeling approach, and how the transfer functions at each port may be derived using a Z-transform technique. Section 5 is devoted to the analysis and discussion of simulation results for the four architectures based on three sets of parameters with high transmission, high reflection and completely symmetric parameters. Optical gain may be used to adjust filter parameters to obtain a desired magnitude response, especially in the frequency range of 1–5 GHz. The spectral response of a structure III device is measured as a function of different injection currents. Conclusions drawn from the simulation and experimental results are given in Section 6.

## 2. Architecture of Photonic Channelizers

**Figure 1.**
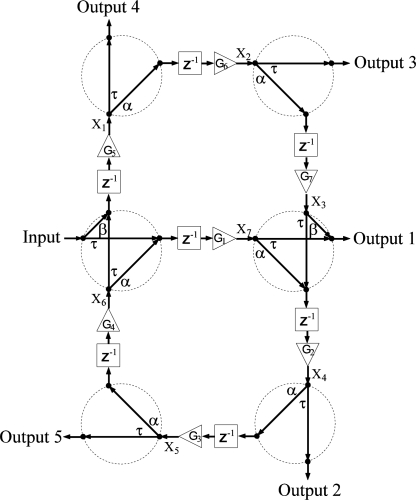
Network diagram of structure I. Transmitted, *τ*, right-directed, *α*, and left-directed, *β*, coupler coefficient components. Photonic channelizer enabled by 6 two-port nano-photonic couplers, *G_i_*(*i* = 1,2,3....) represent gain (triangles), *Z*^−1^ represent unit delay (blocks), and *X_i_* are internal states. Arrows indicate signal flow.

**Figure 2.**
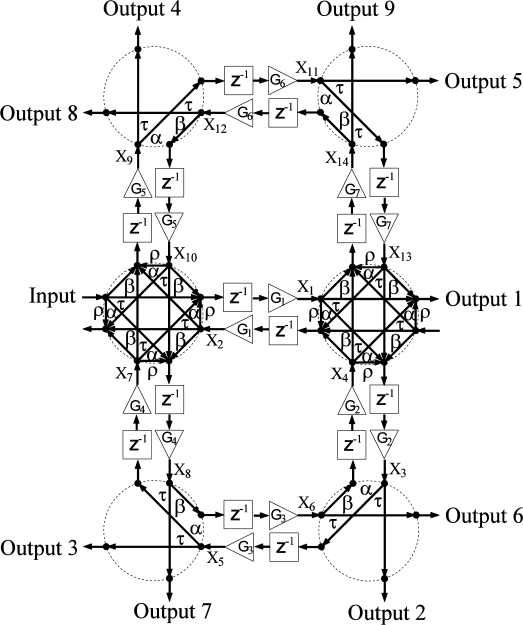
Network diagram of structure II. Transmitted, *τ*, reflected, *ρ*, right-directed,*α*, and left-directed, *β*, coupler coefficient components. Photonic channelizer enabled by 4 two-port couplers and 2 four-port couplers, *G_i_*(*i* = 1,2,3....) represent gain (triangles), *Z*^−1^ represent unit delay (blocks), and *X_i_* are internal states. Arrows indicate signal flow.

**Figure 3.**
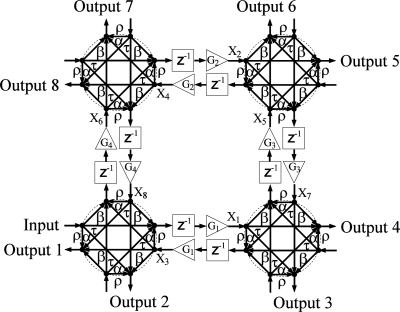
Network diagram of structure III. Transmitted, *τ*, reflected, *ρ*, right-directed,*α*, and left-directed, *β*, coupler coefficient components. Photonic channelizer enabled by 4 four-port couplers, *G_i_*(*i* = 1,2,3....) represent gain (triangles), *Z*^−1^ represent unit delay (blocks), and *X_i_* are internal states. Arrows indicate signal flow.

**Figure 4.**
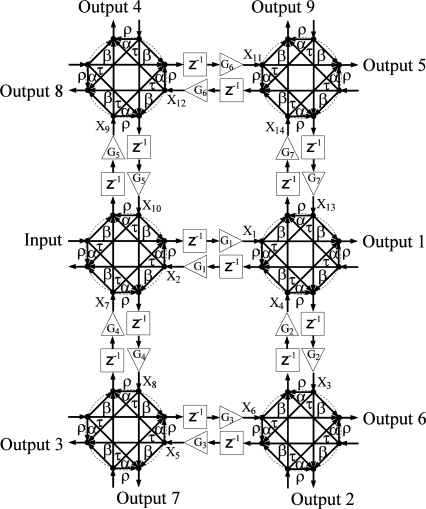
Network diagram of structure IV. Transmitted, *τ*, reflected, *ρ*, right-directed,*α*, and left-directed, *β*, coupler coefficient components. Photonic channelizer enabled by 6 four-port couplers, *G_i_*(*i* = 1,2,3....) represent gain (triangles), *Z*^−1^ represent unit delay (blocks), and *X_i_* are internal states. Arrows indicate signal flow.

The network diagrams of Figures 1–4 show signal flow and directly guide frequency domain algebraic analysis of the filters [16]. The sampling time of the device is defined as the time it takes for a wave to travel the minimum physical distance between adjacent couplers. For this reason, a signal processing technique is utilized based on Z-transform space in describing and modeling the filter structures [17,18]. The operator *Z*^−1^ represents a unit sample delay that is equivalent to a time delay of *nd / c*, where *n* is the refractive index of the waveguide, *d* is the distance between couplers, and *c* is the speed of light in vacuum. Gain elements in these structures represent scaling factors that make each filter an active structure with the ability of reconfiguring frequency response on the order of nanoseconds. In practice, these gain elements are realized by the SOA ridge waveguides that allow movement of filter poles and zeros with injection current, and thus provide higher quality factors for these filters.

A network diagram of a structure based entirely on two-port couplers is shown in Figure 1. The structure I consists of two directional couplers that combine and split an incoming signal into two different waveguides. Physically the two-port coupler is formed by one narrow deep trench oriented 45°to the intersection of two SOA ridge waveguides on InP. Common spacing between couplers defines a constant sampling time in a model description. The recursive nature of the structure categories it as an infinite impulse response (IIR) filter with two simple feedback loops (4^th^ and 6^th^ order) and two feed-forward paths from the input to any output. In total, the structure I consists of 6 two-port couplers, and seven SOAs waveguides. The two-port couplers may couple a signal into multiple waveguides in which the signal is amplified by injection of different currents for different gains. Hence, the possibility of shaping up the frequency response by tuning SOA gains becomes a key step in the design process.

Figure 2 shows the network diagram of an architecture that combines two- and four-port couplers. That is, physically the single trench across each waveguide intersection is replaced by two trenches forming an “X” at an angle of 45˚ with respect to the waveguides. The addition of four-port couplers routes signals into more propagation paths which leads to a higher order filter. In particular, the device is of 14^th^ order with 2^nd^, 4^th^, 6^th^, 8^th^, 10^th^, 12^th^, and 14^th^ order loops. Notice that the second order loops can only be created by using four-port couplers that stem from two consecutive back reflections. Therefore, a single second order loop exists in this structure since only a single waveguide exists between the two four-port couplers in the middle. This becomes of special importance when the case of a channelizer design is discussed with high reflection parameters.

For the network diagram of the third architecture shown in Figure 3, a single loop consisting of 4 four-port couplers with a total of eight inputs/outputs is considered. The structure is similar to that of a traditional optical lattice filter except that the structure has a 2D signal flow due to the existence of scattering parameters *α* and *β*. The structure is of 8^th^ order with even order feedback loops ranging from 2^nd^ to 8^th^ order. Notice that the existence of four 2^nd^ order loops is due to having four consecutive back reflections. This allows a great range of tuning options for poles and zeros of the system.

For the fourth architecture, Figure 4, structure III is extended to include two additional four-port couplers. This extension results in a higher order filter with more gain elements and a more comprehensive signal flow, thus enabling an increase in the tuning range of frequency response. The structure is of 14^th^ order with a total of nine outputs.

## 3. Experiments

Physically the four structures of photonic channelizers shown above may be realized on MQW epitaxial InP wafers. The experimental work here represents a photonic circuit with a highly integrated architecture. The InP epitaxy provides SOA regions between these nano-photonic couplers. These SOAs provide the delay and the broad gain bandwidth for optical signal processing. The speed of these amplifiers provides tremendous agility to the photonic integrated circuit.

The MQW epitaxial structure on 2” Si-doped InP wafers is commercially available from nLight Corporation. The active region consists of three 7.0 nm compressively-strained GaInAsP QWs separated by two 10.0 nm tensile-strained GaInAsP barriers. To fabricate these SOAs on the wafers, ridge waveguides are defined using conventional photolithography and reactive ion etching. High aspect ratio etching of coupler trenches in InP is conducted by focused ion beam patterning and an HBr-based inductively coupled plasma chemistry [14]. Considering processing limitations on trench width, and refractive index of the fill material, trenches filled with alumina by atomic layer deposition [19] have been fabricated and demonstrated. Dielectric isolation and contact definition for the etched InP regions are processed by standard micro-fabrication methods. Select devices are cleaved with ∼1,000 μm lengths from the sample using an automated scribe and break tool.

**Figure 5.**
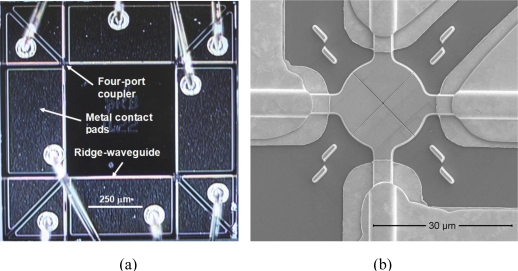
**(a)** Micrograph of a structure III device with 4 four-port nano-photonic couplers at intersections of four ridge-waveguide segments. Gold wire bonds are made to p-type contact pads that uniquely address each waveguide segment. Current may be injected into each SOA ridge waveguide using a common back side n-type contact. **(b)** SEM micrograph of a four-port nano-photonic coupler with metal contacts on waveguides. NPCs have dimensions of 150 nm × 20 μm forming “X” at the intersection of two ridge waveguides. Four rectangular alignment marker pairs adjacent to the circular intersection are used to precisely align NPCs during FIB patterning.

As an example, Figure 5(a) is a micrograph of a structure III type device as-processed. The device may support 8-input and 8-output operation but only 8 SOAs are wired. An injection current applied to metal contact pad along waveguide segment provides gain to an optical signal in each segment. Figure 5(b) shows a scanning electron microscopy (SEM) micrograph of the intersection of four waveguide segments containing a four-port NPC. Two deep trenches are patterned perpendicular to each other and oriented 45° to the ridge waveguides. The 20 μm circular pad forms a broad planar surface at the intersection to facilitate thin and uniform PMMA coating during processing stages of the NPC. Four pairs of rectangular alignment marks placed about the circular pad are used for precision alignment of the NPC with respect to the waveguides.

**Figure 6.**
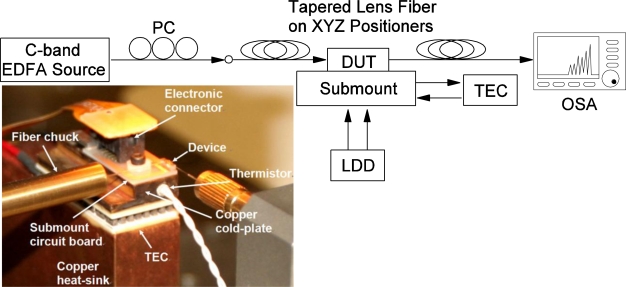
Schematic setup for spectral response measurements of the photonic channelizer devices (EDFA: erbium-doped fiber amplifier, PC: polarization controller, DUT: device under test, LDD: laser diode driver, TEC: thermoelectric cooler, OSA: optical spectrum analyzer). Inset: A custom designed submount and circuit board assembly for device testing.

Figure 6 shows a schematic setup for spectral response measurements of photonic channelizer devices. The inset shows a custom designed submount and circuit board assembly with gold wire ball bonding [Figure 5(a)] from the device to the circuit board for device testing. The submount temperature is controlled by a thermoelectric cooler sandwiched between a copper cold-plate and a heat-sink. Tapered lens fibers are introduced at both input and output ports of the device. A Newport 8000 laser diode driver controller is used to individually drive each waveguide segment through an external electronic connector. The spectral response is recorded by an Agilent 86142B optical spectrum analyzer. All measurements are performed at room temperature.

## 4. Modeling of Photonic Channelizer

### 4.1. State space modeling approach

This section is concerned with the modeling of the four proposed architectures. The main objective of the modeling is to develop a unified method for deriving transfer functions and evaluating the stability of the structures. The state space modeling approach offers a comprehensive analysis of the systems’ internal states which in turn results in better analysis of the filter’s behavior and stability. State space representations [20] are versatile and applicable to diverse types of systems. In addition to enabling a derivation of the transfer matrix, they also provide means of verifying desirable filter features (such as stability) directly in terms of the state space representation. A discrete-time state space representation consists of two sets of equations [21]:

(1) x(k+1)=Ax(k)+Bu(k)

(2) y(k)=Cx(k)+Du(k)

where *x* represents a state of the system, *u* represents an input into the system, and *y* represents an output. Here *x* ∈ *R^n^*, *u* ∈ *R^m^*, *y* ∈ *R^p^*, where *n*, *m*, *p* are the number of states, inputs, and outputs, respectively. In general *m*, *n*, and *p* will not be equal. The states of the system, *X_i_*, may be thought of as latent or hidden variables, which simplify the passage from inputs, *u*, to outputs, *y*, though in several contexts the state variables have concrete physical interpretations. The matrices *A*, *B*, *C*, *D* are *n* × *n*, *n* × *m*, *p* × *n*, and *p* × *m*, respectively. The *A* matrix describes relationship among internal states of the system. The *B* matrix describes the relation between internal states and inputs. The *C* matrix describes relation of internal states with outputs. The *D* matrix describes the direct relation between inputs and outputs. A transfer function having non-zero elements in the *D* matrix (indicating the existence of a direct path from the input to the output) leads to a higher numerator order. The direct path from input to output introduces additional zeros.

### 4.2. Derivation of A, B, C, D matrices for each architecture

In general, the number of states is determined by the number of waveguides in two-port coupler structures and twice the number of waveguides in four-port coupler structures. Given an arbitrary labeling scheme for inputs, outputs, and the states as shown in Figures 1–4, two sets of equations may be derived to construct the state space matrices. For structure I, for example, the first set of equations relating new states with old states and an input can be written as:

(3) $$\begin{array}{l} X_{1}(k+1)=G_{5}\left[{\alpha X}_{6}(k)+\beta u(k)\right]\\ X_{2}(k+1)=G_{6}\left[{\alpha X}_{1}(k)\right]\\ X_{3}(k+1)=G_{7}\left[{\alpha X}_{2}(k)\right]\\ X_{4}(k+1)=G_{2}\left[{\tau X}_{3}(k)+\alpha X_{7}(k)\right]\\ X_{5}(k+1)=G_{3}\left[{\alpha X}_{4}(k)\right]\\ X_{6}(k+1)=G_{4}\left[{\alpha X}_{5}(k)\right]\\ X_{7}(k+1)=G_{1}\left[{\alpha X}_{6}(k)+\tau u(k)\right] \end{array}$$

whereas equations relating output values with respect to current states and an input are given by:

(4) $$\begin{array}{l} Y_{1}(k)={\tau X}_{7}(k)+{\beta X}_{3}(k)\\ Y_{2}(k)={\tau X}_{4}(k)\\ Y_{3}(k)={\tau X}_{2}(k)\\ Y_{4}(k)={\tau X}_{1}(k)\\ Y_{5}(k)={\tau X}_{5}(k) \end{array}$$

The corresponding state space matrices for each architecture are given in the appendix.

### 4.3. Determination of the transfer function

Once the state space matrices are derived, the transfer function matrix is given by:

(5) $$G(z)=C{\left(\mathrm{zI}-A\right)}^{-1}B+D.$$

The transfer function matrix is independent of numbering of the internal states. The (*i^th^*, *j^th^*) entry of *G*(*z*) is the transfer function describing the affect of the *j^th^* input on the *i^th^* output. If one is only interested in the (*i^th^*, *j^th^*) entry, then *G*(*z*) need not be computed entirely. Instead, the *j^th^* column of *B* is multiplied on the left by (*zI* − *A*)^−1^. Then one computes the standard inner product of the resulting vector with *i^th^* row of *C*. To this inner product *D_ij_* is added to find *G_ij_*(*z*). The inverse *z* transform of *G*(*z*) results in an impulse response of the system:

(6) $$H(k)={\mathrm{CA}}^{k-1}B+D\delta (k)$$

System stability has many definitions and types. Traditionally, in the digital signal processing domain, stability is defined as a system in which a bounded input gives a bounded output. The desirable attribute of bounded input-bounded output stability (BIBO) is equivalent to asymptotic stability in the absence of pole-zero cancellations in the following sense: if the system’s transfer matrix is proper (*i.e.*, each entry’s numerator has degree at most equal to that of its denominator) then the system is BIBO stable if each pole of the system has absolute value strictly less than one. Thus, for a variety of practical reasons asymptotic stability of the system is the preferred mode of stability. Hence, we rely on asymptotic stability which dictates that the all eigenvalues of the *A* matrix must have a magnitude less than one, where the eigenvalues represent the poles of the system in the absence of pole-zero cancellations. The eigenvalues of the *A* matrix are computed by solving the characteristic equation det[*zI* − *A*] =0. Further discuss of stability of the systems can be found in our previous work [21,22].

## 5. Results and Discussion

### 5.1. Simulation results

Assuming coupler separation of ∼9 mm by InP waveguides with a refractive index of 3.2, a sampling time of *T_s_* = ∼0.1 nsec or *F_s_* = 10 GHz, a suitable frequency range for RF-photonic signal processing is obtained. In the following examples of channelizer design, lossless, symmetric couplers are assumed which may be characterized by a unitary matrix:

(7) $$S=\left(\begin{array}{llll} \rho & \alpha & \tau & \beta \\ \beta & \rho & \alpha & \tau \\ \tau & \beta & \rho & \alpha \\ \alpha & \tau & \beta & \rho \end{array}\right)$$

In general the state space modeling approach can readily handle imperfections such as lossy couplers. The four-port couplers simulated here are symmetric and without loss, yielding three conditions for energy conservation, based on equal magnitudes of total incident time-average Poynting vectors and total reflected and transmitted time-average Poynting vectors [15,23]:

(8) $$\alpha^{2}+\beta^{2}+\rho^{2}+\tau^{2}=1$$

(9) 2αβ+2ρτ=0

(10) τ(α+β)+ρ(α+β)=0

**Figure 7.**
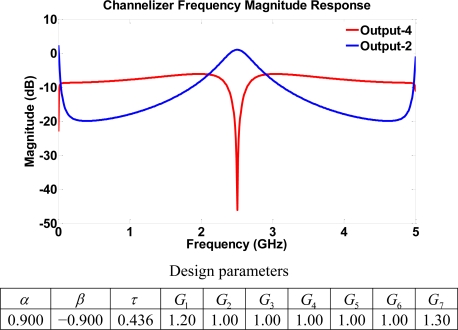
Frequency response for structure I using low transmission parameters.

**Figure 8.**
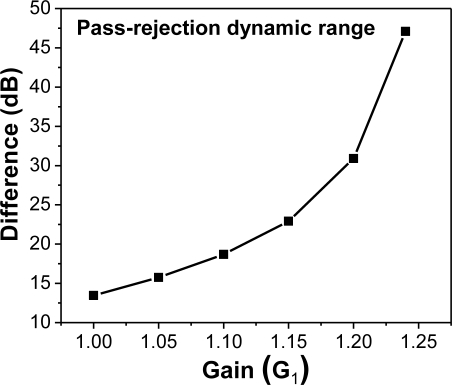
Dynamic range for frequencies (ω = *mπ* + *π*/2), as a function of gain (G_1_) in structure I.

Again, the eigenvalues of computed *A* matrices have a magnitude less than one for all simulation results. Hence, asymptotic stability is ensured in all simulation cases. Channelizer structure I utilizes the frequency responses of output ports with either a bandpass (resonator) or a notch response. This suggests a complementary nature in the output ports that may be used to separate frequencies into multiple bands. Specifically, the notch response notches out frequencies where *ω* = *mπ* + *π*/2 (*m* = 0, 1, 2, 3...), whereas the resonator response passes frequencies where *ω* = *mπ* + *π*/2. Figure 7 depicts a typical frequency response resulting from the Z-transform design and the analysis of structure I, with low transmission parameters. The simulated response of the device shows two outputs, and the transfer function of each output is either a bandpass or a notch filter. The role of the gains comes into play through the dynamic range defined as the difference in dB between the magnitude frequency responses of the pass/attenuate frequencies of interest. The effect of the gains is evident on the channelizer’s dynamic range as increasing the gains enhances the separation between frequencies being (de)emphasized, as shown in Figure 8. Mathematically, the role of gains in the design is to push poles/zeros as close to the unit circle as possible. Hence, the magnitude of notch/peak increases. Similar results for the dynamic range may be obtained by sweeping different gains (e.g., G_7_).

Once the device is fabricated, gains are the only elements that may change the filter’s spectral characteristics. Hence, different manufacturing conditions are considered, and the gains adaptation capabilities are examined. In the design examples below three different sets of parameters are assumed that physically represent three specifications of trench width. Specifically, cases of high transmission, high reflection, and completely symmetric parameters, shown in table [1], satisfying Equations (8–10), are assumed.

**Figure 9.**
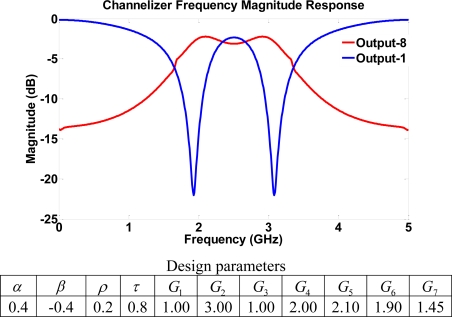
Frequency response for structure II using high transmission parameters. Different notch locations are observed.

Structure I with two feed-forward paths and two feedback loops may only yield a basic channelizer where the range of frequencies for which the notch/resonator peak may be relocated is considered to be limited. More complicated structures with more signals paths (higher order) may result in a more comprehensive channelizer design with more frequency bands. For this reason, the second structure that benefits from the use of 2 four-port couplers is considered for providing a more complicated signal path and a higher order filter. The output ports may also have the same characteristics of bandpass and notch responses as shown in structure I. However, more frequencies can be precisely separated and controlled by adjusting gains. Characteristics of different channelizers may be obtained by using different gain values. Figure 9 shows a notch frequency response with notches at frequencies 1.9 and 3.1 GHz at output 1, whereas the other output port 8 is a bandpass filter with the pass band between 1.9–3.1 GHz. Again, the filter’s dynamic range is greatly affected by current controlled SOA gain values.

**Figure 10.**
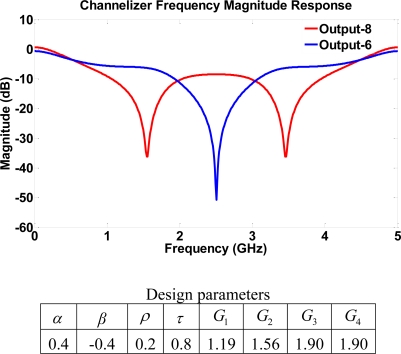
Frequency response for structure III using high transmission parameters. A high dynamic range is achieved.

Next we examine a structure that consists entirely of four-port couplers. The single loop structure has a total of eight bi-directional ports with four waveguides connecting the couplers. Therefore, a total of four gains from SOAs may be used to tune the device. Using the same parameters as in structure II (high transmission) similar results are obtained as shown in Figure 10. Both output port 6 and 8 exhibit a notch frequency response with frequencies 1.8, 2.5, 3.5 GHz being (de)emphasized. The dynamic range in this case may be much higher than in the previous case, which suggests the importance of introducing four-port couplers to provide higher filter order that leads to a better frequency selectivity range.

**Figure 11.**
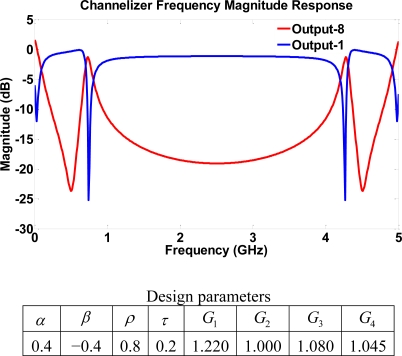
Frequency response for structure III using high reflection parameters.

Let us consider a different manufacturing scenario where a high reflectivity or a wide trench width resulting in low transmission parameters is assumed. In this case the device becomes more sensitive to gain values since the high reflectivity means that the structure may behave like a ring resonator. Mathematically, the poles of the system are greatly affected by the reflection and the scattering coefficients only, due to the absence of transmission coefficients in feedback loops. Thus, transmission components could only affect zeros. That is, the higher the reflection/scattering coefficients, the greater the poles magnitude. However, this may cause stability issues if the gains are not properly chosen. Hence, it is important to investigate the asymptotic stability for cases of high reflectivity as it does not take high gain values for the system to become unstable. This fact explains the high gain values that the device can handle in cases of high transmission parameters. Still with slightly low gain values, a higher dynamic range is achieved than with the case of unity gains. The output ports have notch filters with different notch frequency locations, as shown in Figure 11, for structure III using high reflection parameters.

**Figure 12.**
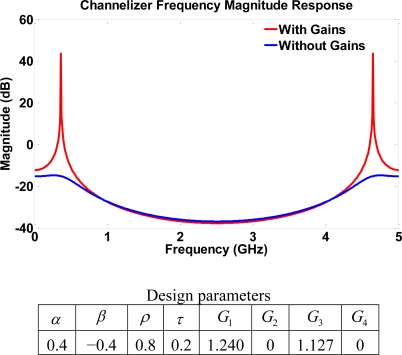
Frequency response of output 4 for structure III with some unused gains, resulting in a resonator behavior.

Another potential application is to explore the resulting frequency responses from switching off some of the gains in the structure. Generally, a higher order filter may be preferred over lower order filters to achieve better performance. However, the higher order structure implies a higher number of SOAs which may degrade the optical signal to noise ratio. Therefore, the lower order structures may still be the designer’s first choice for some cases that require simple frequency separation channels with a moderate dynamic range. It is thus useful to obtain a lower filter order through the switching property of SOAs. Specifically, an obvious resonator behavior may be obtained by switching off *G*_2_ and *G*_4_ in Figure 3. This leads to a new structure layout of 4^th^ order where the feedback loops become more dependent on reflection coefficients. In particular, an all-pole system is exhibited by some of the output ports when switching off *G*_2_ and *G*_4_. The primary role of the gains is to push the poles as close as possible to the unit circle. All the output port responses have the same behavior which is very similar to a sharp resonator. Therefore, only frequencies with $\omega =\frac{2}{3}\pi m+\frac{\pi }{8}$ are emphasized, and the effect of the gains is shown in Figure 12. One drawback of this technique is the weakening and loss of some output signals due to the signal absorption by the un-amplified SOAs. For instance, note that both output 7 and 8 are shut off in structure III.

**Figure 13.**
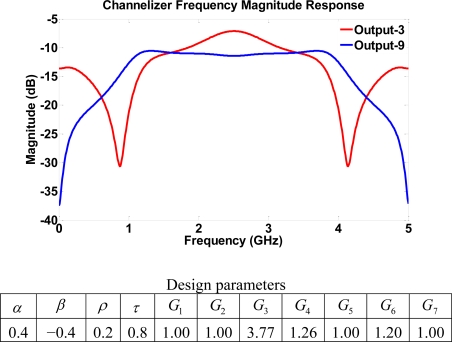
Frequency response for structure IV using high transmission parameters.

Structure IV is obtained by extending structure III with two additional four-port couplers in the vertical direction. The frequency responses of all outputs can be either bandpass with wide passbands or notch filters with different notch locations. The increased number of gain stages allows for a variety of tunability of notch locations. Figure 13 indicates a channelizer with notches at 0.85 and 4.1 GHz on output 3 whereas output 9 has a bandpass response with a passband between 0.89 and 4.05 GHz.

**Figure 14.**
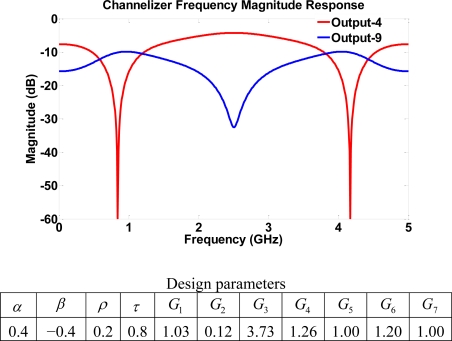
Frequency response for structure IV using high transmission parameters.

**Figure 15.**
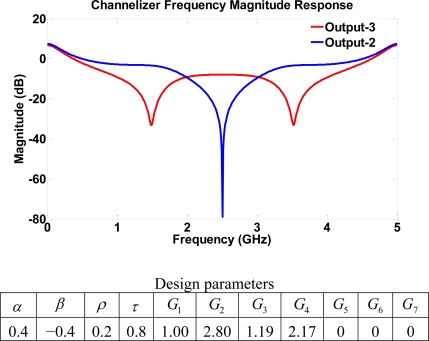
Frequency response for structure IV using high transmission parameters with different combination of unused gains.

A variety of different notch locations are obtained by using a combination of parameters with and without gains. The choice of the off gain modifies the layout of the structure. Figure 14 shows the frequency response with notches at 0.84, 2.50, 4.17 GHz, whereas Figure 15 shows frequency notches at 1.48, 2.50, 3.52 GHz. This indicates a channelizer structure that may be simply designed using the single loop building block of structure III since switching off gains *G*_5_, *G*_6_, and *G*_7_ results in the same structure with only 4 four-port coupler elements. The major setback for going from a more dense structure to a lower one is the complete loss of some output port signals. For example, switching off the gain stages *G*_5_, *G*_6_, and *G*_7_ leads to the loss of outputs 4, 5, 8 and 9. That is, the scaled structure will have less output than that of structure III itself. The dynamic range achieved using structure IV is considered to be the highest among all four structures. This results from a large pool of poles and zeros of the system.

**Figure 16.**
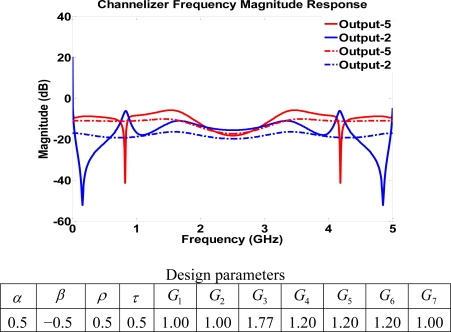
Frequency response using symmetric parameters for structure IV. Solid lines represent output signals with gains. Dashed lines represent output signals with unity gains.

It is practical to consider a manufacturing case with symmetric parameters in which an incoming signal is coupled equally in four different directions. In the symmetric case, the gains have an increased role in shaping frequency response by breaking up the symmetry. Figure 16 shows how gains may be used to change the behavior of output ports 5 and 2 of structure IV, which is similar to creating a complementary behavior.

From the previous simulation results we may conclude that structure IV offers the most comprehensive frequency tuning options. A variety of frequency bands may be separated within the free spectral range for structure IV. While structure I may provide a proper dynamic range, its ability to distinguish different frequencies within the free spectral range is very limited. This is a direct result from the simple signal flow and the limited number of poles and zeros that the structure provides. Significant improvements are noted when migrating to structures II and III.

Modeling of the proposed structures through the state space approach is very practical and may provide solutions for accurate analysis of the system’s asymptotic stability. The sparse nature of the *A*, *B*, *C*, *D* matrices suggests easily recognizable patterns when expanding an existing structure. Hence, automated generation of the state space matrices is achievable. Any of the four structures may be easily extended by concatenating more couplers and waveguides, thus creating more frequency tuning options. For example, consider the extension of structure III by connecting duplicate blocks of the same structure in a matrix fashion, *i.e.*, a 2 × 2 structure indicates a connection of four structure III or 16 four-port couplers. Given an arbitrary number of rows and columns of structure III, the total number of states or the filter’s order is determined by *P* = *N* × *M*, where *P*, *N*, and *M* are number, columns, and rows of structure III, respectively. *X* = *P*×8+*N*×[(*M*−1)×4]+*M*×[(*N*−1)×4], where *X* is number of states. The corresponding number of input/outputs is given by *M* × 4 + *N* × 4. For instance, assume an extension model of size 2 × 2. This implies a filter of 48^th^ order, with 16 input/outputs. With any arbitrary labeling scheme, filling in these state space matrices is easily obtained by using simple algorithms. Also, the expanded systems may still be reduced to the original system block, if needed, using the switching property of the SOAs.

### 5.2. Experimental results

**Figure 17.**
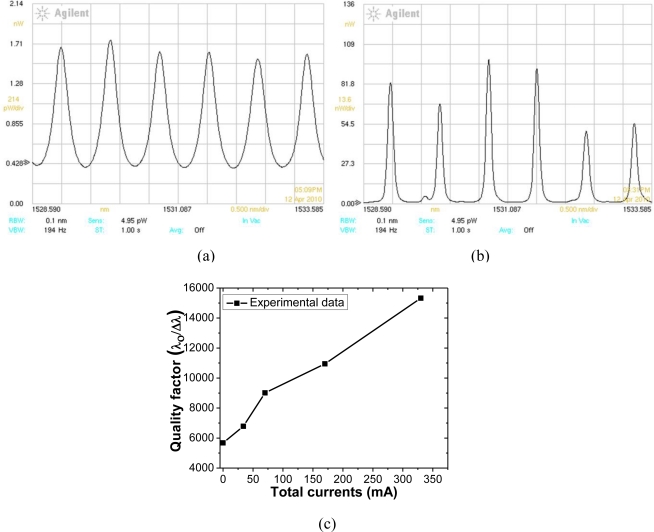
**(a)** Spectral response of a structure III type device with a total driving current of 34 mA for *G*_1_, *G*_2_, *G*_3_, and *G*_4_ gain stages. **(b)** Spectral response of a structure III device with a total driving current of 170mA for *G*_1_, *G*_2_, *G*_3_, and *G*_4_ gain stages. **(c)** Measurement of quality factor as a function of total driving current (*λ_o_* : peak wavelength, Δ*λ* : spectral width of one peak at FWHM).

Figures 17(a) and (b) show spectral responses measured from output 2 of a structure III type device with a total driving current of 34 mA and 170 mA for *G*_1_, *G*_2_, *G*_3_, and *G*_4_ gain stages, respectively. As the total applied current is increased, the quality factor of the device is improved significantly, as shown in Figure 17(c).

This indicates that the quality factor is controllably tuned by application of an optical gain by current injection. Similar results are also obtained from an optical tapped-delay-line microwave signal processor filter, and its passband width may be tuned by controlling the gain of an active erbium-doped fiber [24]. The experiments shown here are for a photonic channelizer configured with the coupler separation of ∼500 μm. The device is thus targeted for applications in a RF frequency range of 90 GHz. The current work for realization of an active photonic channelizer may be formed on one single semiconductor chip. This minimal footprint component improves yield and conserves real estate in a wide variety of optical systems/integrated optical material systems.

## 6. Conclusions

A theoretical study of RF-photonic channelizers with four architectures formed by active integrated filters with tunable gains is presented. The four proposed architectures vary in the structural layout and internal nano-photonic coupler formations, (either two-port or four-port). The behavior of the nano-photonic coupler is experimentally based on the phenomenon of frustrated total internal reflection. These photonic channelizers may be fabricated in one single semiconductor chip on MQW epitaxial InP wafers using conventional microelectronics processing techniques.

A state space modeling approach is used to derive the transfer functions and analyze the stability of these filters, and the *A*, *B*, *C*, *D* matrices are demonstrated for each architecture. Stability may be determined from the eigenvalues of *A* matrix in the state space representations. Three different manufacturing scenarios are assumed, and the gains are used as adaptive elements to provide the necessary frequency responses of the channelizers. Our simulation results indicate that different realizations may have a remarkable impact on each filter’s performance primarily in terms of each channelizer’s frequency range and dynamic range. The characteristic bandpass and notch filter responses of the outputs of each structure are the basis of channelizer architecture. Structure IV offers the most comprehensive frequency tuning options compared to the other structures. Structure I only provide a proper dynamic range with limited ability of frequency distinction. Structures II and III achieve significant improvements in the middle.

As a starting point, the measurement of spectral response shows the enhancement of quality factor for a structure III type device by using higher injection currents that provide higher optical gains. These compact active filters on integrated photonic circuits with MQW InP-based technology hold considerable potential for channelizer applications. Fabrication of a photonic channelizer with coupler separation of ∼9 mm for a 10 GHz sampling frequency is currently underway. Simulation results shown here will be studied.

## Figures and Tables

**Table 1.** Coupler coefficients used for the three proposed manufacturing cases.

Design	**α**	**β**	**ρ**	**τ**
High transmission	0.4	−0.4	0.2	0.8
High reflection	0.4	−0.4	0.8	0.2
Symmetric	0.5	−0.5	0.5	0.5

## Appendix

State space matrices for each structure are extracted from the two basic sets of equations in (1).

**State space matrices for structure I**

$$A_{1}=\left[\begin{array}{ccccccc} 0 & 0 & 0 & 0 & 0 & G_{5}\alpha & 0\\ G_{6}\alpha & 0 & 0 & 0 & 0 & 0 & 0\\ 0 & G_{7}\alpha & 0 & 0 & 0 & 0 & 0\\ 0 & 0 & G_{2}\tau & 0 & 0 & 0 & G_{2}\alpha \\ 0 & 0 & 0 & G_{3}\alpha & 0 & 0 & 0\\ 0 & 0 & 0 & 0 & G_{4}\alpha & 0 & 0\\ 0 & 0 & 0 & 0 & 0 & G_{1}\alpha & 0 \end{array}\right]$$

$$B_{1}=\left[\begin{array}{c} G_{5}\beta \\ 0\\ 0\\ 0\\ 0\\ 0\\ G_{1}\tau \end{array}\right]$$

$$C_{1}=\left[\begin{array}{ccccccc} 0 & 0 & \beta & 0 & 0 & 0 & \tau \\ 0 & 0 & 0 & \tau & 0 & 0 & 0\\ 0 & \tau & 0 & 0 & 0 & 0 & 0\\ \tau & 0 & 0 & 0 & 0 & 0 & 0\\ 0 & 0 & 0 & 0 & \tau & 0 & 0 \end{array}\right]$$

$$D_{1}=\left[0\right]$$

**State space matrices for structure II**

$$A_{2}=\left[\begin{array}{cccccccccccccc} 0 & G_{1}\rho & 0 & 0 & 0 & 0 & G_{1}\alpha & 0 & 0 & G_{1}\beta & 0 & 0 & 0 & 0\\ G_{1}\rho & 0 & 0 & G_{1}\beta & 0 & 0 & 0 & 0 & 0 & 0 & 0 & 0 & G_{1}\alpha & 0\\ G_{2}\alpha & 0 & 0 & G_{2}\rho & 0 & 0 & 0 & 0 & 0 & 0 & 0 & 0 & G_{2}\tau & 0\\ 0 & 0 & 0 & 0 & 0 & G_{2}\beta & 0 & 0 & 0 & 0 & 0 & 0 & 0 & 0\\ 0 & 0 & G_{3}\alpha & 0 & 0 & 0 & 0 & 0 & 0 & 0 & 0 & 0 & 0 & 0\\ 0 & 0 & 0 & 0 & 0 & 0 & 0 & G_{3}\beta & 0 & 0 & 0 & 0 & 0 & 0\\ 0 & 0 & 0 & 0 & G_{4}\alpha & 0 & 0 & 0 & 0 & 0 & 0 & 0 & 0 & 0\\ 0 & G_{4}\beta & 0 & 0 & 0 & 0 & G_{4}\rho & 0 & 0 & G_{4}\tau & 0 & 0 & 0 & 0\\ 0 & G_{5}\alpha & 0 & 0 & 0 & 0 & G_{5}\tau & 0 & 0 & G_{5}\rho & 0 & 0 & 0 & 0\\ 0 & 0 & 0 & 0 & 0 & 0 & 0 & 0 & 0 & 0 & 0 & G_{5}\beta & 0 & 0\\ 0 & 0 & 0 & 0 & 0 & 0 & 0 & 0 & G_{6}\alpha & 0 & 0 & 0 & 0 & 0\\ 0 & 0 & 0 & 0 & 0 & 0 & 0 & 0 & 0 & 0 & 0 & 0 & 0 & G_{6}\beta \\ 0 & 0 & 0 & 0 & 0 & 0 & 0 & 0 & 0 & 0 & G_{7}\alpha & 0 & 0 & 0\\ G_{7}\beta & 0 & 0 & G_{7}\tau & 0 & 0 & 0 & 0 & 0 & 0 & 0 & 0 & G_{7}\rho & 0 \end{array}\right]$$

$$B_{2}=\left[\begin{array}{c} G_{1}\tau \\ 0\\ 0\\ 0\\ 0\\ 0\\ 0\\ G_{4}\alpha \\ G_{5}\beta \\ 0\\ 0\\ 0\\ 0\\ 0 \end{array}\right]$$

$$C_{2}=\left[\begin{array}{cccccccccccccc} \tau & 0 & 0 & \alpha & 0 & 0 & 0 & 0 & 0 & 0 & 0 & 0 & \beta & 0\\ 0 & 0 & \tau & 0 & 0 & 0 & 0 & 0 & 0 & 0 & 0 & 0 & 0 & 0\\ 0 & 0 & 0 & 0 & \tau & 0 & 0 & 0 & 0 & 0 & 0 & 0 & 0 & 0\\ 0 & 0 & 0 & 0 & 0 & 0 & 0 & 0 & \tau & 0 & 0 & 0 & 0 & 0\\ 0 & 0 & 0 & 0 & 0 & 0 & 0 & 0 & 0 & 0 & \tau & 0 & 0 & 0\\ 0 & 0 & 0 & 0 & 0 & \tau & 0 & 0 & 0 & 0 & 0 & 0 & 0 & 0\\ 0 & 0 & 0 & 0 & 0 & 0 & 0 & \tau & 0 & 0 & 0 & 0 & 0 & 0\\ 0 & 0 & 0 & 0 & 0 & 0 & 0 & 0 & 0 & 0 & 0 & \tau & 0 & 0\\ 0 & 0 & 0 & 0 & 0 & 0 & 0 & 0 & 0 & 0 & 0 & 0 & 0 & \tau \end{array}\right]$$

$$D_{2}=\left[0\right]$$

**State space matrices for structure III**

$$A_{3}=\left[\begin{array}{cccccccc} 0 & 0 & G_{1}\rho & 0 & 0 & 0 & 0 & G_{1}\beta \\ 0 & 0 & 0 & G_{2}\rho & 0 & G_{2}\alpha & 0 & 0\\ G_{1}\rho & 0 & 0 & 0 & 0 & 0 & G_{1}\alpha & 0\\ 0 & G_{2}\rho & 0 & 0 & G_{2}\beta & 0 & 0 & 0\\ G_{3}\beta & 0 & 0 & 0 & 0 & 0 & G_{3}\rho & 0\\ 0 & 0 & G_{4}\alpha & 0 & 0 & 0 & 0 & G_{4}\rho \\ 0 & G_{3}\alpha & 0 & 0 & G_{3}\rho & 0 & 0 & 0\\ 0 & 0 & 0 & G_{4}\beta & 0 & G_{4}\rho & 0 & 0 \end{array}\right]$$

$$B_{3}=\left[\begin{array}{cccccccc} G_{1}\alpha & 0 & 0 & 0 & G_{1}\tau & 0 & 0 & 0\\ 0 & 0 & G_{2}\beta & 0 & 0 & 0 & G_{3}\tau & 0\\ 0 & G_{1}\beta & 0 & 0 & 0 & G_{1}\tau & 0 & 0\\ 0 & 0 & 0 & G_{2}\alpha & 0 & 0 & 0 & G_{2}\tau \\ 0 & G_{3}\tau & 0 & 0 & 0 & G_{3}\alpha & 0 & 0\\ G_{4}\tau & 0 & 0 & 0 & G_{4}\beta & 0 & 0 & 0\\ 0 & 0 & 0 & G_{3}\tau & 0 & 0 & 0 & G_{3}\beta \\ 0 & 0 & G_{4}\tau & 0 & 0 & 0 & G_{4}\alpha & 0 \end{array}\right]$$

$$C_{3}=\left[\begin{array}{cccccccc} 0 & 0 & \tau & 0 & 0 & 0 & 0 & \alpha \\ 0 & 0 & \beta & 0 & 0 & 0 & 0 & \alpha \\ \alpha & 0 & 0 & 0 & 0 & 0 & \tau & 0\\ \tau & 0 & 0 & 0 & 0 & 0 & \beta & 0\\ 0 & \tau & 0 & 0 & \alpha & 0 & 0 & 0\\ 0 & \beta & 0 & 0 & \tau & 0 & 0 & 0\\ 0 & 0 & 0 & \alpha & 0 & \tau & 0 & 0\\ 0 & 0 & 0 & \tau & 0 & \beta & 0 & 0 \end{array}\right]$$

$$D_{3}=\left[\begin{array}{cccccccc} \beta & 0 & 0 & 0 & \rho & 0 & 0 & 0\\ \rho & 0 & 0 & 0 & \alpha & 0 & 0 & 0\\ 0 & \rho & 0 & 0 & 0 & \beta & 0 & 0\\ 0 & \alpha & 0 & 0 & 0 & \rho & 0 & 0\\ 0 & 0 & 0 & \beta & 0 & 0 & 0 & \rho \\ 0 & 0 & 0 & \rho & 0 & 0 & 0 & \alpha \\ 0 & 0 & \rho & 0 & 0 & 0 & \beta & 0\\ 0 & 0 & \alpha & 0 & 0 & 0 & \rho & 0 \end{array}\right]$$

**State space matrices for structure IV**

$$A_{4}=\left[\begin{array}{cccccccccccccc} 0 & G_{1}\rho & 0 & 0 & 0 & 0 & G_{1}\alpha & 0 & 0 & G_{1}\beta & 0 & 0 & 0 & 0\\ G_{1}\rho & 0 & 0 & G_{1}\beta & 0 & 0 & 0 & 0 & 0 & 0 & 0 & 0 & G_{1}\alpha & 0\\ G_{2}\alpha & 0 & 0 & G_{2}\rho & 0 & 0 & 0 & 0 & 0 & 0 & 0 & 0 & G_{2}\tau & 0\\ 0 & 0 & G_{2}\rho & 0 & 0 & G_{2}\beta & 0 & 0 & 0 & 0 & 0 & 0 & 0 & 0\\ 0 & 0 & G_{3}\alpha & 0 & 0 & G_{3}\rho & 0 & 0 & 0 & 0 & 0 & 0 & 0 & 0\\ 0 & 0 & 0 & 0 & G_{3}\rho & 0 & 0 & G_{3}\beta & 0 & 0 & 0 & 0 & 0 & 0\\ 0 & 0 & 0 & 0 & G_{4}\alpha & 0 & 0 & G_{4}\rho & 0 & 0 & 0 & 0 & 0 & 0\\ 0 & G_{4}\beta & 0 & 0 & 0 & 0 & G_{4}\rho & 0 & 0 & G_{4}\tau & 0 & 0 & 0 & 0\\ 0 & G_{5}\alpha & 0 & 0 & 0 & 0 & G_{5}\tau & 0 & 0 & G_{5}\rho & 0 & 0 & 0 & 0\\ 0 & 0 & 0 & 0 & 0 & 0 & 0 & 0 & G_{5}\rho & 0 & 0 & G_{5}\beta & 0 & 0\\ 0 & 0 & 0 & 0 & 0 & 0 & 0 & 0 & G_{6}\alpha & 0 & 0 & G_{6}\rho & 0 & 0\\ 0 & 0 & 0 & 0 & 0 & 0 & 0 & 0 & 0 & 0 & G_{6}\rho & 0 & 0 & G_{6}\beta \\ 0 & 0 & 0 & 0 & 0 & 0 & 0 & 0 & 0 & 0 & G_{7}\alpha & 0 & 0 & G_{7}\rho \\ G_{7}\beta & 0 & 0 & G_{7}\tau & 0 & 0 & 0 & 0 & 0 & 0 & 0 & 0 & G_{7}\rho & 0 \end{array}\right]$$

$$B_{4}=\left[\begin{array}{c} G_{1}\tau \\ 0\\ 0\\ 0\\ 0\\ 0\\ 0\\ G_{4}\alpha \\ G_{5}\beta \\ 0\\ 0\\ 0\\ 0\\ 0 \end{array}\right]$$

$$C_{4}=\left[\begin{array}{cccccccccccccc} \tau & 0 & 0 & \alpha & 0 & 0 & 0 & 0 & 0 & 0 & 0 & 0 & \beta & 0\\ 0 & 0 & \tau & 0 & 0 & \alpha & 0 & 0 & 0 & 0 & 0 & 0 & 0 & 0\\ 0 & 0 & 0 & 0 & \tau & 0 & 0 & \alpha & 0 & 0 & 0 & 0 & 0 & 0\\ 0 & 0 & 0 & 0 & 0 & 0 & 0 & 0 & \tau & 0 & 0 & \alpha & 0 & 0\\ 0 & 0 & 0 & 0 & 0 & 0 & 0 & 0 & 0 & 0 & \tau & 0 & 0 & \alpha \\ 0 & 0 & \beta & 0 & 0 & \tau & 0 & 0 & 0 & 0 & 0 & 0 & 0 & 0\\ 0 & 0 & 0 & 0 & \beta & 0 & 0 & \tau & 0 & 0 & 0 & 0 & 0 & 0\\ 0 & 0 & 0 & 0 & 0 & 0 & 0 & 0 & \beta & 0 & 0 & \tau & 0 & 0\\ 0 & 0 & 0 & 0 & 0 & 0 & 0 & 0 & 0 & 0 & \beta & 0 & 0 & \tau \end{array}\right]$$

$$D_{4}=\left[0\right]$$
